# Humans exhibit associative symmetry in the absence of backward training and stimulus overlap

**DOI:** 10.1002/jeab.70020

**Published:** 2025-05-06

**Authors:** Victor M. Navarro, Edward A. Wasserman

**Affiliations:** ^1^ Cardiff University Cardiff UK; ^2^ The University of Iowa Iowa City IA USA

**Keywords:** associative symmetry, backward, bidirectional, forward, stimulus equivalence

## Abstract

A recent survey of the evidence on associative symmetry in humans revealed that nearly all the demonstrations either unintentionally trained backward stimulus pairings and/or had a temporal overlap between the stimuli being trained. We consider these criticisms and improve on our own method of “associative networks.” In this method, participants learn multiple stimulus pairings via arbitrary matching‐to‐sample tasks in which the stimuli are concurrently presented as sample and comparison stimuli. In Experiment 1, human participants learned a bidirectional network (in which symmetry was synergistic) and a unidirectional network (in which symmetry was antagonistic) or two unidirectional networks (removing explicit reinforcement of backward stimulus pairings). In Experiment 2, participants learned two unidirectional networks; however, we removed the temporal overlap between sample and comparison stimuli by imposing a 1‐s delay between them. Both experiments showed robust evidence of symmetry, suggesting that the expression of symmetry in humans survives the most common confounds in published research.

Some 4 decades ago, Murray Sidman and his colleagues began a research program to systematically characterize how organisms learn to treat nominally distinct stimuli as functional equivalents (a research story later detailed in Sidman, 1994), a phenomenon known as stimulus equivalence. Sidman advanced three principles to establish stimulus equivalence: reflexivity, symmetry, and transitivity. Although each principle has gathered a great deal attention throughout the decades (Eilifsen & Arntzen, 2021; Minster et al., 2006; Sidman et al., 1989; Sidman & Tailby, 1982; Urcuioli & Swisher, 2012), it is (associative) symmetry that motivates the present work.

Associative symmetry occurs when the learning produced by a directional stimulus pairing results in subjects expressing the stimulus pairing in the opposite direction. Note that the notion of direction can denote both spatiotemporal and/or hierarchical properties of the stimuli. In the spatiotemporal case, take, for example, a subject who has learned via Pavlovian conditioning that the presentation of stimulus A is followed by the occurrence of stimulus B (A → B, the forward pairing). In this case, the subject might not only produce responses indicating that they expect B during or shortly after the presentation of A but also produce responses indicating that they expect A during or shortly after the presentation of B, even though the backward pairing (B → A) was never explicitly trained. The hierarchical case is best exemplified using conditional discriminations, in which the reinforcement contingencies of some stimuli are determined by other stimuli. Take, for example, a subject who has learned via operant conditioning that the reinforcement contingencies of stimuli A and B are determined by stimuli X and Y such that responses to A are reinforced if A was preceded by X but not Y (X → A+/Y → A‐) and responses to B are reinforced if B was preceded by Y but not X (Y → B+/X → B‐). In this case, the subject might not only differentially respond to A and B in accord with the contingencies stated above but also might respond more to X when preceded by A than by B and respond more to Y when preceded by B than by A, even though these swapped stimulus contingencies (A → X+/A → Y‐ and B → X‐/B → Y+) were never explicitly trained.

In the laboratory, symmetry is often assessed via arbitrary matching tasks (Sidman et al., 1982) meant to teach the conditional discriminations of the kind we described above. In these tasks, subjects must choose the correct comparison stimulus upon presentation of a sample stimulus. For example, experimenters might first teach subjects to choose A but not B after being presented with X (X|A + B‐) and to choose B but not A after being presented with Y (Y|A‐B+). Typically, soon after subjects attain a performance criterion, they are given probe trials in which the roles of the stimuli are reversed, with A and B being presented as samples and X and Y being presented as comparisons (A|X?Y? and B|X?Y?). If subjects choose X over Y after being presented with A (and Y over X after being presented with B), then subjects are said to have shown evidence for associative symmetry, suggesting that the stimulus contingencies established after initial training (X → A+ and Y → B+^1^) have effectively produced control in the opposite direction (A → X+ and B → Y+) or have established control in a nondirectional manner (A + ↔X+, B + ↔Y+).

The principle of symmetry has enjoyed a great deal of attention in both behavior analysis and cognitive research communities, as it is believed to be the vehicle for acquiring symbol–referent associations during language learning (Imai et al., 2021; Schmidt et al., 2022; Sidman, 2008). Most critically, the difference in the ease and flexibility with which human and nonhuman animals express symmetry has been interpreted by some as an apparent discontinuity between the cognitive abilities of humans and nonhumans (Galizio & Bruce, 2018; Kabdebon & Dehaene‐Lambertz, 2019).

Sidman et al. (1982) first failed to find evidence of symmetry in monkeys and baboons, and things have not changed much since then. Surveys of the literature suggest that successful demonstrations of symmetry in nonhumans have been very difficult to obtain, with associative symmetry being the exception rather than the rule (Galizio & Bruce, 2018; Lionello‐DeNolf, 2009, 2021), although recent methodological innovations are promising (Galizio et al., 2023; Mason et al., 2021).

Several of the successful demonstrations of associative symmetry in nonhuman animals suggest that some additional *associative scaffolding* sometimes needs to be in place for symmetry to be expressed. For example, Frank and Wasserman (2005) assessed the development of associative symmetry in pigeons using a successive arbitrary matching task (e.g., X → A+, X → B‐, Y → A‐, and Y → B+) containing clip art images of natural objects and gave some subjects concurrent identity‐matching training (e.g., X → X+, X → Y‐, Y → Y+, Y → X‐, A → A+, A → B‐, B → B+, B → A‐). Their results were unequivocal (and replicated later by Swisher & Urcuioli, 2015, and Urcuioli, 2008; but see Bruce et al., 2022, and Prichard et al., 2015); pigeons given concurrent identity training were likelier to exhibit associative symmetry than pigeons that did not (see also Tomonaga et al., 1991, for similar findings in chimpanzees).

In a tour de force, Urcuioli (2008) presented a fruitful theory of equivalence class formation and a thorough analysis of the conditions leading to the expression of associative symmetry in pigeons. Urcuioli suggested that the identity training that Frank and Wasserman (2005) gave their pigeons might have been the scaffolding they required to express symmetry (see also Campos et al., 2014, for a counterintuitive result using oddity training). He argued that pigeons might treat the same nominal stimulus (e.g., A) as functionally different stimuli depending on whether it appears as a sample (A1) or a comparison (A2) on any given trial. He reasoned then that the reinforcement contingencies learned via arbitrary matching (e.g., X1 → A2+) are not observed during symmetry tests (e.g., A1 → X2?) because the sample during tests (A1) is not the same as the comparison used on arbitrary matching trials (A2). Urcuioli thus reasoned that identity training would help bridge the different versions of A by forming an equivalence class containing A as a sample and A as a comparison (A1 → A2), which could then be integrated with the rest of a larger equivalence class established via arbitrary matching (e.g., A1 → A2 → X1 → X2).

Urcuioli (2008) also made a similar case for the spatial location of samples and comparisons during two‐alternative arbitrary matching tasks (see also Jones & Elliffe, 2013, for a discussion of stimulus location in matching and signal detection tasks). In those tasks, a sample is followed by the presentation of two comparisons (e.g., X → A + B‐, Y → A‐B+), often in spatial locations (e.g., left or right) that differ from the location of the sample (e.g., center). As it has been shown that stimulus location can strongly influence stimulus generalization in the pigeon (Lea et al., 2022; Lionello & Urcuioli, 1998), traditional two‐alternative matching tasks could yield equivalence classes that must be bridged for subjects to express symmetry. The addition of identity training in a two‐alternative setting (e.g., cX → lX + rY‐, where c, l, and r denote central, left, and right locations, respectively) should provide such a scaffolding, but, surprisingly, doing so does not readily yield symmetry in pigeons (Lionello‐DeNolf & Urcuioli, 2002; Urcuioli, 2008). Therefore, the conditions under which pigeons express associative symmetry in two‐alternative matching tasks remain elusive.

The difficulty in obtaining associative symmetry in nonhuman animals (Galizio & Bruce, 2018; Lionello‐DeNolf, 2009, 2021) has persuaded some researchers to propose that the spontaneous development of symmetry might be a uniquely human phenomenon (Imai et al., 2021; Kabdebon & Dehaene‐Lambertz, 2019). However, Chartier and Fagot (2022) have recently suggested that the high success rate for demonstrations of symmetry in humans is due to researchers accidentally providing participants with the scaffolding necessary to express symmetry. After reviewing 37 studies investigating the emergence of symmetry in humans, Chartier and Fagot concluded that nearly all of them had methodological shortcomings across two broad categories. First, most human studies have used tasks in which the samples and comparison stimuli appear with complete or partial temporal overlap. Such overlap could help bridge the temporal gap inherent to sequentially presented stimuli or, more directly, might have resulted in the explicit training of bidirectional pairings. Second, many human studies used instructions containing bidirectional language, which might have prompted participants to actively ignore stimulus location and order of presentation.

In Navarro and Wasserman (2020; one of the studies reviewed by Chartier and Fagot), we developed a novel task to study the emergence of associative symmetry in humans and pigeons. The task used two‐alternative, simultaneous, arbitrary matching trials to teach subjects two different “associative networks.” To exploit the potential benefits of multiple‐exemplar training (Schusterman & Kastak, 1993), each network contained 16 nonoverlapping conditional discriminations involving pexigrams (P) and objects (O): visual stimuli that could be both samples and comparisons (Figure 1A). In the bidirectional network, any given sample was the correct comparison stimulus when its own correct comparison was presented as a sample (e.g., O1|P1 + P2‐ and P1|O1 + O2‐, O2|P2 + P1‐ and P2|O2 + O1‐, etc.). However, such bidirectionality did not hold in the unidirectional network (e.g., O5|P5 + P7‐ but P5|O8 + O5‐, and O6|P6 + P5‐ but P6|O5 + O6‐). It is worth noting that these associative networks were identical across several factors. First, each network had an equal number of objects and pexigrams, thus supporting the same number of positive contingencies to be learned. Second, each stimulus in each network was presented equally often as a sample and a comparison, avoiding novelty issues that might arise from training stimuli as samples/comparisons first and only later probing them in their opposite role. Finally, the comparisons in each network were equally likely to be presented as the incorrect and correct comparison, thus having roughly the same marginal probability of reinforcement when considered in isolation.^2^

**FIGURE 1:**
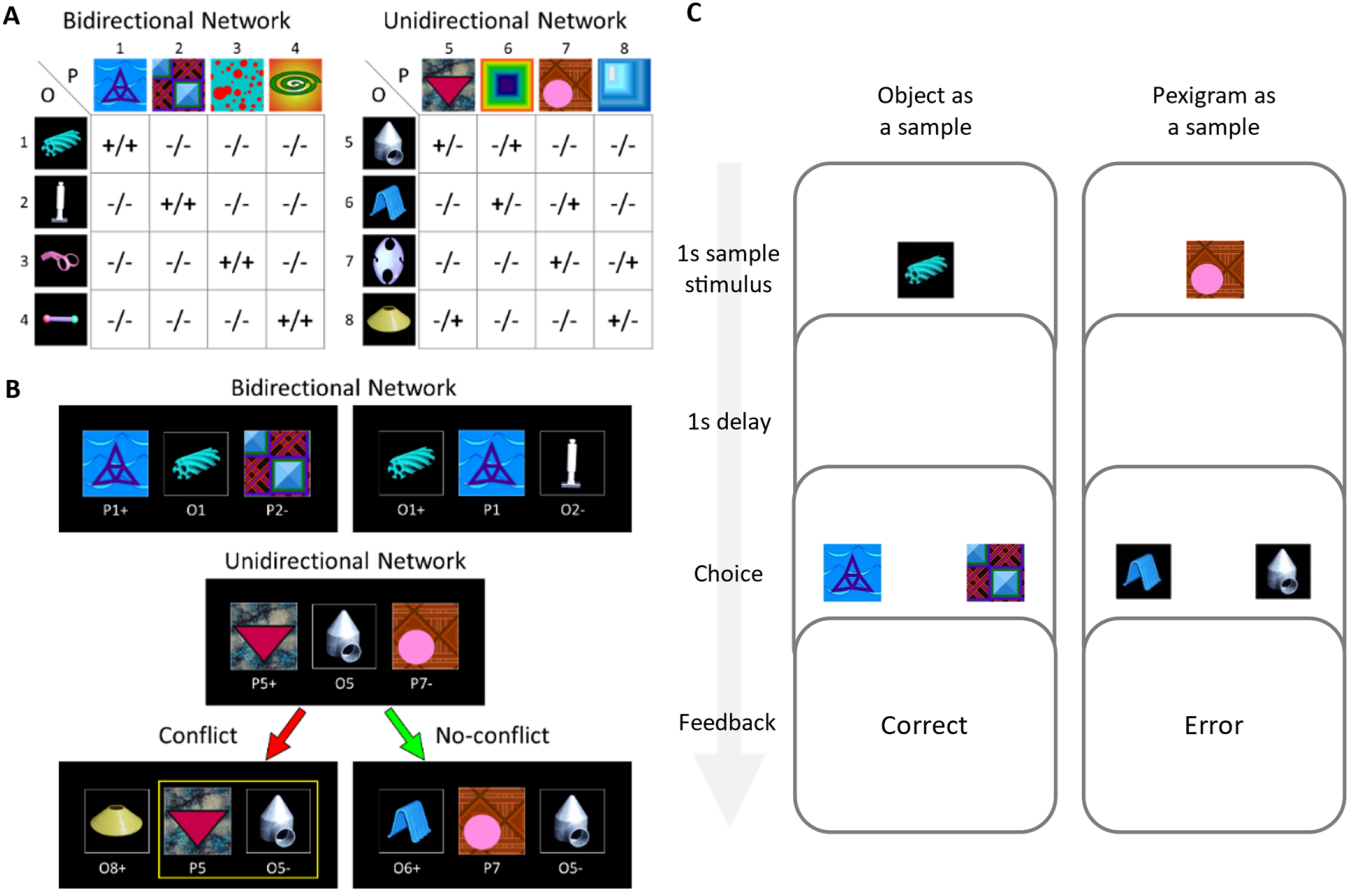
Experimental design and trial structure. Panel A: Abbreviated bidirectional (left) and unidirectional (right) networks used in Navarro and Wasserman (2020). The first and second sign in each 4 × 4 matrix entry specifies whether the comparison stimulus is correct (+) or incorrect (−) on “Object as a sample” and “Pexigram as a sample” trials, respectively. The experiments presented here used trials from two, 6 × 6 matrices. Panel B: Exemplary trials from bidirectional and unidirectional networks. For each stimulus triad, the stimulus in the center is the sample and the two side stimuli are the comparisons (+ and ‐ for positive and negative comparisons, respectively). The location of positive and negative comparisons was randomized. See the main text for details. Panel C: Schematic of the two types of 2AFC trials used in Experiment 2: “Object as sample” and “Pexigram as a sample.”

Our first prediction was straightforward: If subjects formed the equivalence classes conducive to associative symmetry, then they should respond more accurately (or learn more quickly) in the bidirectional network than in the unidirectional network because the expression of symmetry was synergistic only in the former. For example, O1|P1 + P2‐ trials will result in learning of the O1|P1+ contingency and perhaps might also result in learning the reverse, P1|O1+ contingency, thereby benefiting performance in P1|O1 + O2‐ trials. However, if subjects were to express symmetry when faced with the unidirectional network, then their accuracy should receive no such benefit. The O5|P5 + P7‐ trials might still result in learning both O5|P5+ and P5|O5+ contingencies; however, we had arranged the second of those contingencies to be explicitly incorrect (i.e., punished) via P5|O8 + O5‐ trials. Our prediction of better performance on the bidirectional network than the unidirectional network held for humans but not for pigeons (cf. Bidirectional and No‐conflict trials in Figure 2).

**FIGURE 2:**
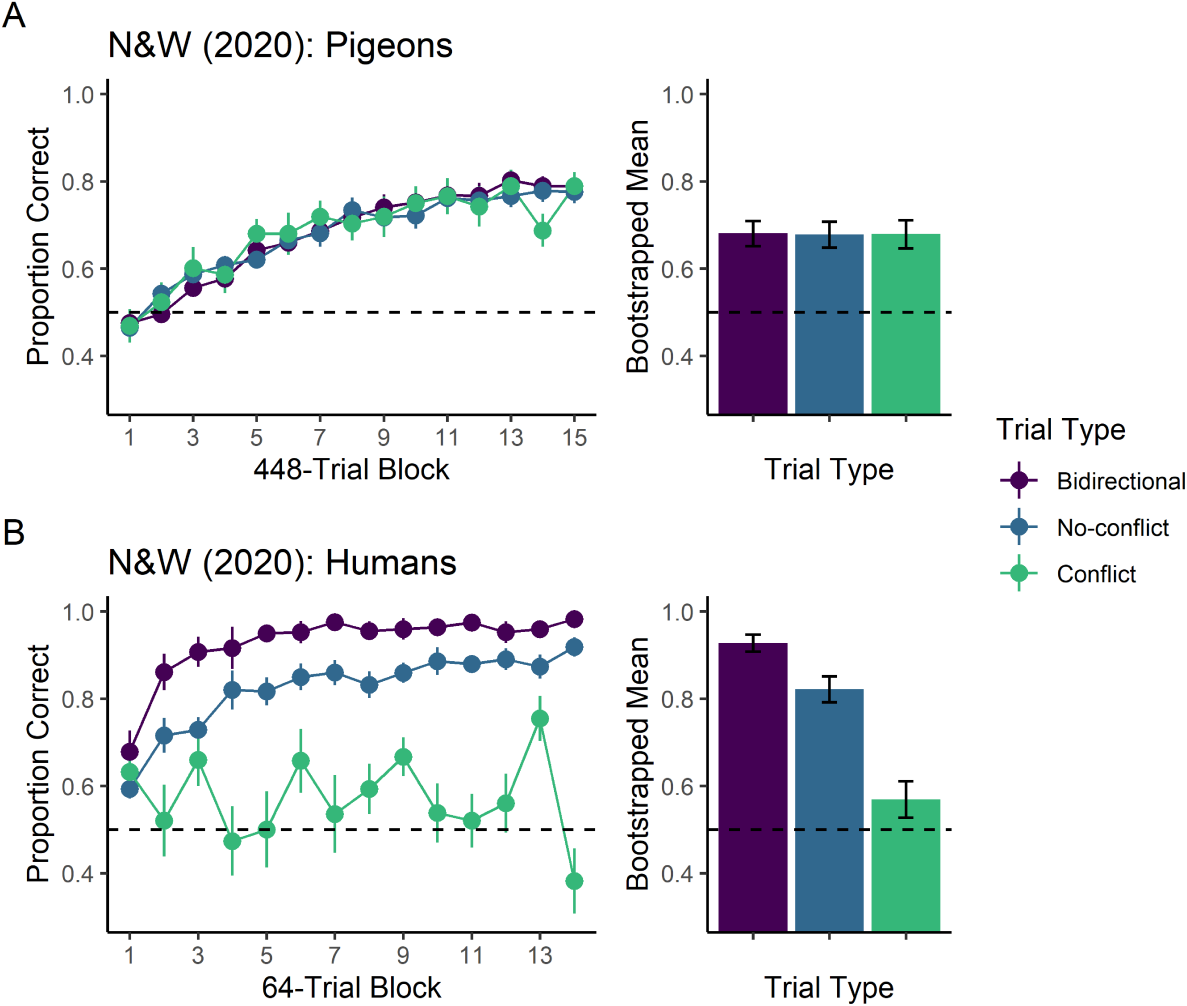
Navarro and Wasserman (2020) results. Panel A: Results for four pigeons. Panel B: Results for 12 humans. Across rows, left panels show the proportion of correct choices per trial type (bidirectional, no‐conflict, and conflict) as a function of trial blocks; error bars represent the standard error of the mean. The right panels show bootstrap estimates of the arithmetic mean proportion of correct choices for each trial type; error bars represent 95% CI of the bootstrapped samples.

Our second prediction concerned two distinct populations of trials within the unidirectional network. On “conflict” trials, subjects expressing symmetry faced a comparison that was incorrect on that trial but that had a positive contingency with the sample on other trials (Figure 1B, yellow rectangle). Such was the case for the P5|O5+ contingency, which might have been (incidentally) established via O5|P5 + P7‐ trials but which we arranged to be nonreinforced via P5|O8+ O5‐ trials. In “no‐conflict” trials, however, bidirectional learners faced a comparison stimulus that had a negative contingency with the sample on trials in which their roles were reversed. For example, subjects ought to perform well on P7|O6 + O5‐ trials regardless of whether they express symmetry because O5 was never the correct comparison for sample P7. Again, our prediction of better performance on no‐conflict trials versus conflict trials held only for humans, not for pigeons (cf. Conflict and No‐conflict trials in Figure 2).

There are many reasons for our failure to detect performance indicative of symmetry in pigeons (see Navarro & Wasserman, 2020, for a discussion). However, as Chartier and Fagot (2022) suggest, perhaps we were too hasty in taking our human results as indicative of associative symmetry. Although our original study contained task instructions that never mentioned the nature of the bidirectional network (and thus lacked biasing language), we sought to obtain more convincing demonstrations of symmetry in human participants by addressing two shortcomings in our original study.

First, we wanted to show that concurrent training with a bidirectional network was unnecessary for our participants to show greater accuracy on no‐conflict than conflict trials. Although this was not one of the methodological shortcomings noted by Chartier and Fagot (2022), the trials from the bidirectional network might have encouraged participants' use of a general strategy (or stimulus encoding) that might be difficult to distinguish from associative symmetry. For example, a strategy such as “stimuli go in pairs such that when one is presented in the middle of the screen, the other stimulus to the side of it will be correct” would lead to poor accuracy on conflict trials, just as real associative symmetry would. Thus, in Experiment 1 we trained a group of participants with one bidirectional and one unidirectional network (BI‐UN group) and another group of participants with two unidirectional networks (UN‐UN group). Having only unidirectional networks in the UN‐UN group might discourage those participants from adopting bidirectional strategies.

Second, we wanted to show that temporal overlap between sample and comparison stimuli was unnecessary for our participants to express associative symmetry. In our original design, the sample stimulus was followed by two comparison stimuli. Still, all three stimuli remained on the screen until our participants responded, and this temporal overlap might have taught our participants an equivalence class that ignores the role of the stimuli, facilitating the expression of symmetry. Thus, in Experiment 2, we trained a single group of subjects with two unidirectional networks, but this time, there was a 1‐s delay between the offset of the sample and the onset of the comparison stimuli. As in our original study, the critical question in both experiments was whether accuracy on conflict trials would be lower than on no‐conflict trials.

## METHOD

### Participants

Participants were recruited via Amazon's Mechanical Turk platform, compensated US$8 for their participation and were awarded a bonus of $3 if their accuracy scores were above the mean of the experimental cohort. Only users located in the United States were eligible to participate. Forty participants took part in Experiment 1. Participants had a mean age of 44.95 years (*SD* = 12.86), and 25/40 participants identified as female. Participants in this experiment were randomly assigned to one of two groups (“BI‐UN” or “UN‐UN,” each with *n* = 20). Twenty participants participated in Experiment 2. Participants had a mean age of 45.42 years (*SD* = 10.86), and 7/20 participants identified as female. All procedures were according to the Declaration of Helsinki and were approved by the Institutional Review Board at The University of Iowa (IRB ID 201808798).

### Stimuli

The stimulus set comprised 12 pictures of objects that were hard to name by humans and 12 complex color patterns that we termed “pexigrams.” See Figure 1 for some sample images. Because the participants completed the tasks on their personal computers, the final size of the stimuli could not be controlled. Instead, the stimuli were dynamically scaled so they were identical in size and could be shown inline. The identity of each stimulus and its location in the associative networks was randomized on a participant‐by‐participant basis.

### Procedure

#### Consent, instructions, and debriefing

After participants accepted the task, they were given a hyperlink to the online task, programmed using the jsPsych library (de Leeuw, 2015). Upon access to the webpage, participants were welcomed with a consent form explaining the nature of the task, their compensation, and their rights as participants. The consent form only described the topography of the task (e.g., “observing pictures on the screen,” “pressing buttons,” etc.). Once participants consented to participate in the study, they were presented with unbiased task instructions (Appendix A). Participants were then quizzed via three multiple‐option questions (Appendix B, after Le Pelley et al., 2019) and commenced the task after correctly answering those three questions (failure to do so required them to reread the instructions and redo the quiz). Upon completion of the task, participants were debriefed on the purposes of the study.

#### Learning task

Participants completed a total of 480 trials, organized across two 240‐trial blocks. Within each block, we balanced the frequency of occurrence for each sample (six samples per network), each sample's unique trials (five per sample), locations of correct and incorrect comparisons (left or right), stimulus roles (sample or comparison), and networks (two different networks). In Experiment 1, participants had to learn both bidirectional and unidirectional networks (group BI‐UN) or two unidirectional networks (group UN‐UN). All participants in Experiment 2 had to learn two unidirectional networks.

On each trial, a sample stimulus was presented for 1 s and its correct and incorrect comparisons were presented alongside (Experiment 1) or after a 1‐s delay since the sample had disappeared from the screen (Experiment 2; see Figure 1C). The correct comparison was presented on every trial, but the incorrect comparison varied randomly from trial to trial. The comparison stimuli remained on the screen until participants chose one of the comparisons by pressing the “F” or “J” keys on their keyboards (for selecting the left and right comparisons, respectively). Choice of the correct and incorrect comparisons resulted in a “Correct” or “Error message,” respectively, displayed for 1 s, and the next trial started immediately afterwards. Participants were told their average accuracy over a self‐paced break given every 120 trials.

### Data analysis

The data sets, model files, and the scripts used to run the analyses presented here are accessible at https://osf.io/8qk4e/. All analyses and visualizations were carried out using R (R Development Core Team, 2021) and the *dplyr* (Wickham et al., 2022), *ggplot2* (Wickham et al., 2021), *patchwork* (Pedersen, 2020), *report* (Makowski et al., 2022), and *lme4* (Bates et al., 2022) packages. We assessed the reliability of the effects observed on a group‐by‐group basis by estimating logistic mixed‐effects models on participant responses (correct and incorrect responses coded as 1 and 0, respectively), including block (1 to 8, centered on the last block) and trial type (bidirectional, no‐conflict, and conflict trials, with the no‐conflict trials as the reference level) as fixed effects. The random‐effects structure of each model consisted of participant‐level effects on group‐level parameters. Random effects were selected via model comparison. Starting from a model containing only participant intercepts, we added participant‐level effects and compared model fit via χ^2^ tests, using α < .05 as a criterion for retaining the more complex model. For fixed effects, we report coefficient estimates, *b* (log odds of making a correct response), their 95% confidence intervals, and their statistical significance (against the null hypothesis of *b* = 0).

## RESULTS

### Experiment 1

**FIGURE 3:**
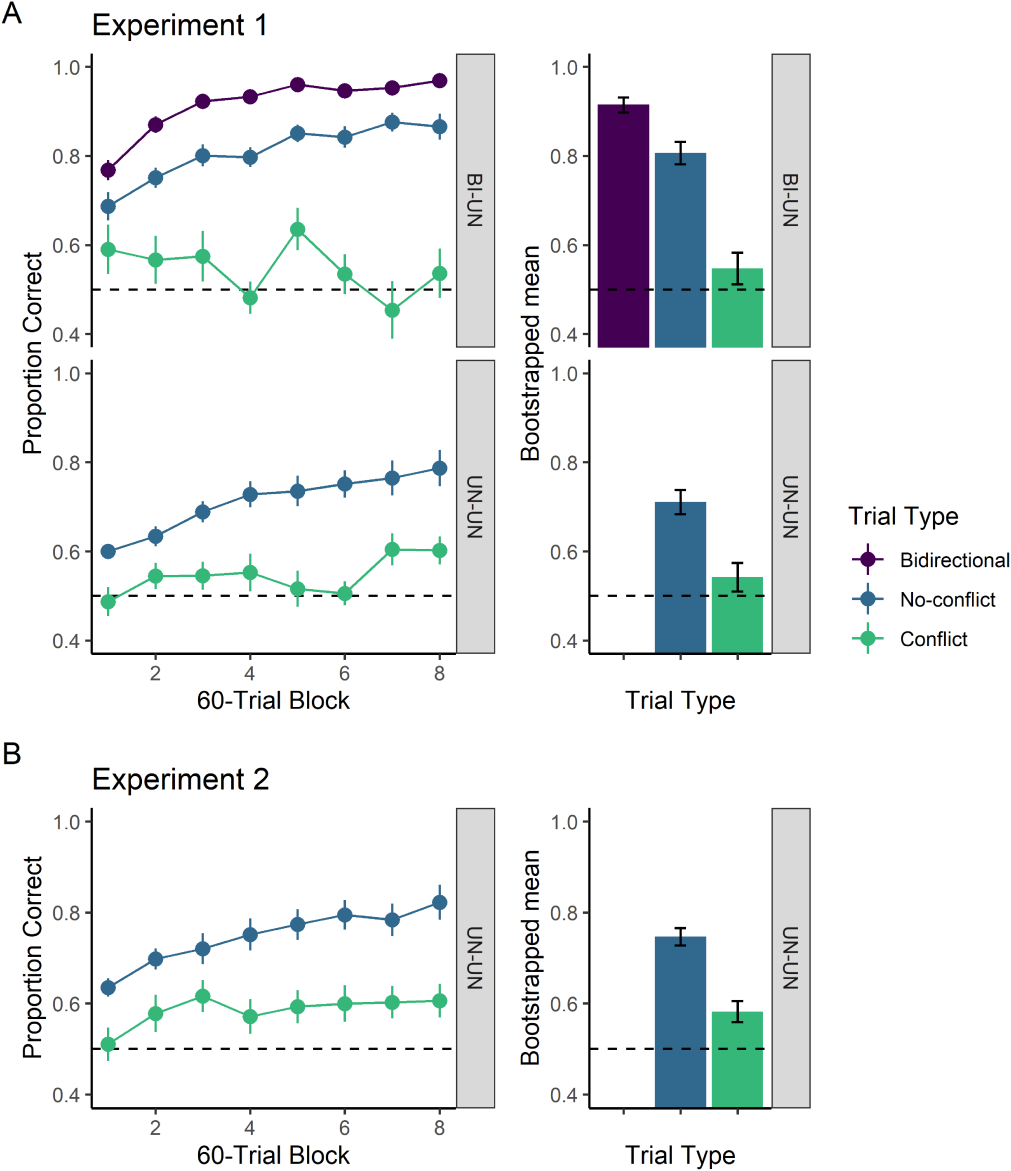
Experimental results. Panel A: Results from Experiment 1 (simultaneous presentation of sample and comparison stimuli). Panel B: Results from Experiment 2 (1‐s delay between sample offset and comparisons onset). Across rows, left panels show the proportion of correct choices per trial type (bidirectional, no‐conflict, and conflict) as a function of trial blocks; error bars represent the standard error of the mean. The right panels show bootstrap estimates of the arithmetic mean proportion of correct choices for each trial type; error bars represent 95% CI of the bootstrapped samples.

The left panels in Figure 3A show the proportion of correct choices per trial type as a function of training blocks for the BI‐UN (top) and UN‐UN (bottom) groups. Relative to their performance on no‐conflict trials, participants in the BI‐UN group learned faster on bidirectional trials (*b* = 0.18, 95% CI [0.12, 0.25], *p* < .001) but slower on conflict trials (*b* = −0.22, 95% CI [−0.29, −0.16], *p* < .001). As in Navarro and Wasserman (2020), the overall frequency of bidirectional trials was higher than that of no‐conflict trials (5 to 4). At the same time, the frequency of no‐conflict trials was much higher than that of conflict trials (4 to 1). To deal with these numerical disparities, we generated 10,000 bootstrapped estimates for the mean proportion of correct responses on each trial type via stratified sampling. To do so, we sampled (with replacement) 16 trials for each participant and trial type. As the top‐right panel in Figure 3A shows, the ordering observed in the learning functions was maintained for overall accuracy: Participants were the most accurate on bidirectional trials (*M* = 0.92, 95% CI = [0.90, 0.93]), followed by no‐conflict trials (*M* = 0.81, 95% CI = [0.78, 0.83]), and conflict trials (*M* = 0.55, 95% CI = [0.51, 0.58]).

Most critically, for this experiment, accuracy of the UN‐UN group on no‐conflict trials was greater than that on conflict trials (Figure 3A, bottom row), even though that group was never trained with bidirectional trials. Specifically, during training, participants in the UN‐UN group were slower in learning to respond accurately on conflict trials (*b* = −0.09, 95% CI [−0.13, −0.04], *p* < .001) than on no‐conflict trials. Again, bootstrapped estimates revealed that the trial ordering observed during training mapped onto the overall accuracy for each trial; the choices of the UN‐UN group were more accurate on no‐conflict trials (*M* = 0.71, 95% CI = [0.68, 0.74]) than on conflict trials (*M* = 0.54, 95% CI = [0.51, 0.57]).

**FIGURE 4:**
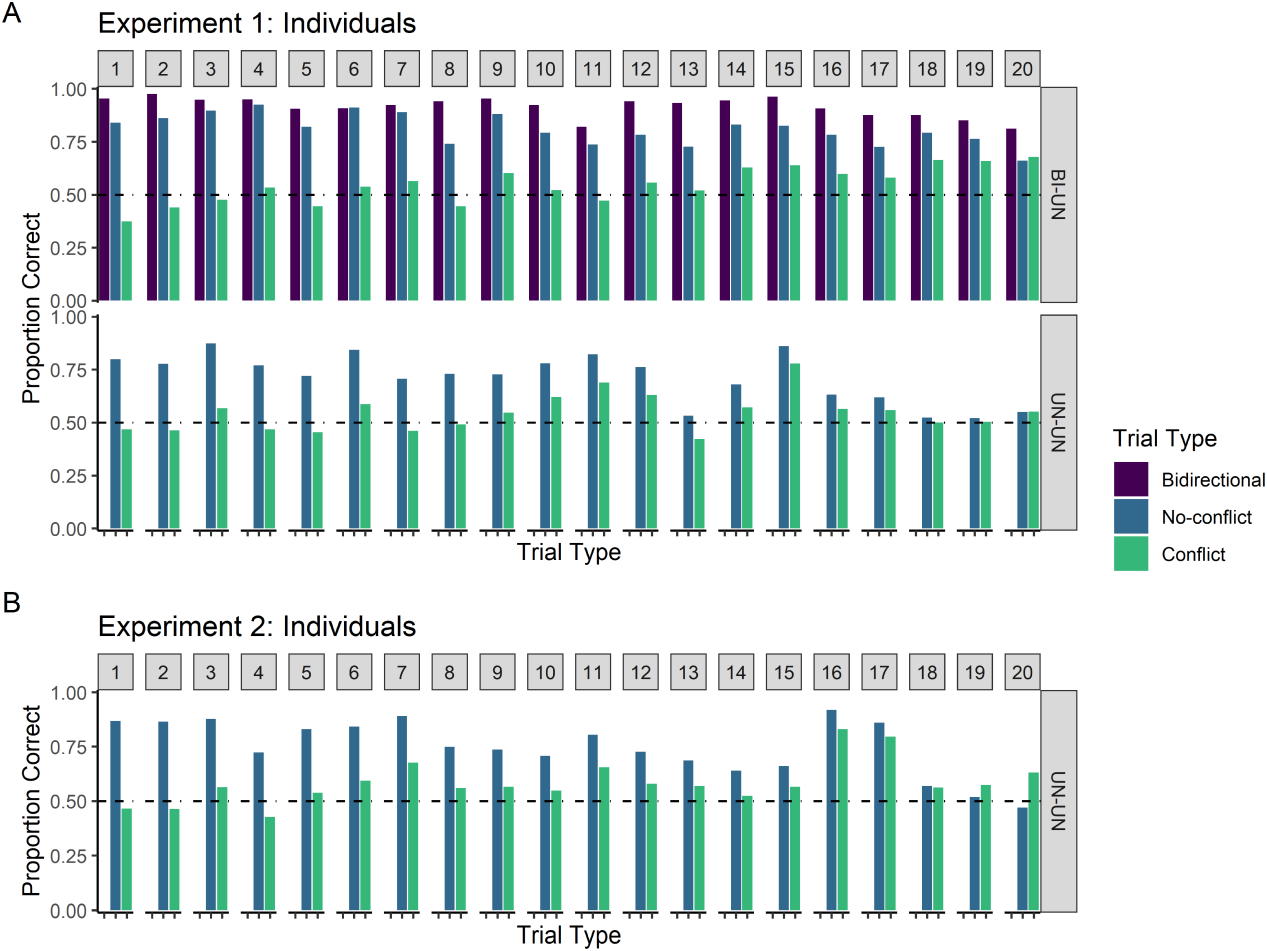
Individual participants' results. Panel A: Results from Experiment 1 (simultaneous presentation of sample and comparison stimuli). Panel B: Results from Experiment 2 (1‐s delay between sample offset and comparisons onset). Across rows, participants within each group are sorted in descending order from the greatest to the smallest difference between each participant's proportion of correct responses on no‐conflict and conflict trials.

Figure 4A shows the performance of each participant in Experiment 1 on each of the trial types available to them. We ranked participants within each group according to the difference in their proportion of correct responses on no‐conflict and conflict trials (which can be loosely interpreted as a proxy of associative symmetry). Thus, within each group, Participant 1 shows the largest difference in favor of no‐conflict trials, whereas Participant 20 shows the smallest (or sometimes the opposite difference). Notably, all but one of the participants in the BI‐UN group (participant 20, top‐right panel of Figure 4A) showed performance in line with symmetry. The expression of symmetry was weaker in the UN‐UN group, with several participants who did not show a difference also performing the task poorly (e.g., 18, 19, 20). A closer look at these data revealed greater difference scores in the BI‐UN group (median = 0.27) than in the UN‐UN group (median = 0.15). Despite this disparity, 19/20 participants in each group had a difference score greater than zero, attesting to the robustness with which symmetry was expressed in each condition despite potential differences in overall task difficulty.

### Experiment 2

As Figure 3B indicates, the 1‐s delay between the sample and comparison stimuli arranged in Experiment 2 did not prevent the expression of symmetry. An analysis of the learning curves showed that participants learned more slowly on conflict trials than on no‐conflict trials (*b* = −0.13, 95% CI [−0.18, −0.08], *p* < .001). Again, the bootstrapped estimates of overall performance clearly reflected the ordering observed during training (*M* = 0.58, 95% CI = [0.56, 0.61] for conflict trials and *M* = 0.75, 95% CI = [0.73, 0.77] for no‐conflict trials).

A comparison of the experiments suggests that introducing the 1‐s delay between the sample and comparisons resulted in better overall performance (cf. UN‐UN groups in Figure 3A and B). An analysis of the bootstrapped samples revealed that this was indeed the case. Although not by much, participants in Experiment 2 were slightly more accurate on both no‐conflict (*M* = 0.04, 95% CI = [0.002, 0.07]) and conflict trials (*M* = 0.04, 95% CI = [0.001, 0.08]) than participants in the UN‐UN group in Experiment 1 were. Note also how the size of this effect is identical for both trial types (0.04). There are no a priori reasons for the delay having this general effect. If a delay disrupts bidirectional encoding, then such a disruption should increase accuracy on conflict trials but not affect no‐conflict trials. The size of this effect is small and might reflect an insufficient sample size (e.g., there is a transient disruption in accuracy across Blocks 5 and 6 in the bottom‐left panel of Figure 3A); we thus leave explorations of this effect for future investigative efforts.

Figure 4B shows the performance of individual participants sorted by the difference between their performance on no‐conflict and conflict trials. Much like participants in the UN‐UN group in Experiment 1, participants with low (or negative) difference scores tended also to have poor task performance. Notably, the distribution of difference scores in Experiment 2 was quite similar to that observed for the UN‐UN group in Experiment 1. The median difference score was also 0.15 this time, with 18 of 20 participants having difference scores greater than zero.

## DISCUSSION

The present experiments replicated and addressed two shortcomings in the methods used by Navarro and Wasserman (2020): (1) the concurrent training of backward stimulus pairings in the BI network given to the BI‐UN participants and (2) the temporal overlap between the sample and comparison stimuli in the arbitrary matching trials given to subjects. In Experiment 1, we trained a group of participants (UN‐UN) with two unidirectional networks in which forward and backward stimulus pairings were explicitly trained in one direction only (e.g., O5|P5 + P7‐ but P5|O8 + O5‐; Figure 1). The UN‐UN group's accuracy on no‐conflict trials was greater than in conflict trials, demonstrating the emergence of associative symmetry (Figure 3A, lower panel). As these participants had no experience with a bidirectional network, their poorer performance on “backward” conflict trials than on no‐conflict trials must have been due to the expression of a stimulus class learned primarily via “forward” trials. In Experiment 2, we extended these results by introducing a 1‐s delay between the offset of the sample stimulus and the onset of the comparison stimuli, a manipulation we reasoned might disrupt the formation of symmetry by establishing sequential stimulus relations (e.g., Romaniuk & Williams, 2000). Even then, the participants in Experiment 2 showed as much evidence of associative symmetry as participants in the UN‐UN group from Experiment 1 (cf. Panels A and B in Figure 3). Clearly, a temporal overlap between the sample and comparison stimuli was not a necessary condition for the expression of associative symmetry.

What could be the reasons behind the robustness with which humans express associative symmetry? One possibility is the degree of abstraction with which nominal stimuli are processed by the human brain (Galizio & Bruce, 2018; Swisher & Urcuioli, 2015). It has been documented that the control established via matching tasks can be complex, with attentional processes modulating the exact stimulus features that gain behavioral control (Stromer et al., 1993). Some have argued that the depth and hierarchical nature of the human visual stream allow for the extraction of stimulus features at different levels of abstraction (Bracci & Op De Beeck, 2023) and the availability of more abstract stimulus features that do not depend on incidental stimulus properties such as location or scale will enable humans' responding to come under the control of task‐relevant features (e.g., stimulus identity; see Beurms et al., 2017) instead of task‐irrelevant ones (e.g., stimulus location; see Swisher & Urcuioli, 2015).

If we deem nonhumans such as pigeons to be models of incidental stimulus control, then how can we make humans more pigeon‐like? One way to do so would be to make incidental features of the stimulus part of the task itself, thus making different versions of the same nominal stimulus more discriminable (i.e., functionally distinct). For example, one might arrange arbitrary stimulus relations contingent on stimulus location or participant responses (García & Benjumea, 2006). If such a task results in human participants encoding these additional aspects of the stimulus, then their ability to express symmetry might be weakened.

A second reason for the robustness of symmetry in humans might be that they can extract complete information from each two‐alternative matching trial. In discussing the reasons for demonstrations of symmetry being more likely in successive than in simultaneous arbitrary matching, Urcuioli (2008) noted that successive preparations explicitly program nonreinforcement for the negative comparisons with the same frequency that they program reinforcement for the positive comparisons. Of course, simultaneous preparations cannot enforce similar constraints, as the frequency of nonreinforcement is inversely related to overall accuracy. Urcuioli thus reasoned that the greater frequency of nonreinforced sample–comparison combinations in successive matching tasks would lead to the formation of stimulus classes based on nonreinforced stimulus combinations, making classes based on reinforced stimulus combinations more discriminable and therefore making the expression of symmetry more likely, similar to a differential outcomes effect (Trapold, 1970; Urcuioli, 2005). Critically, we explicitly told participants that one of the comparisons was correct for the sample on each trial in both of our experiments, and participants might have used this information covertly to retrospectively deduce the contingency of the unselected comparison. If so, every trial (regardless of its outcome) might have promoted the learning of both reinforced and nonreinforced stimulus classes, thus facilitating the expression of symmetry. Note, however, that it should be possible to weaken symmetry by introducing additional comparisons on every trial: Under such conditions, the contingencies experienced after selecting one comparison cannot be used to infer the contingencies prevailing for the other comparisons.

In conclusion, humans readily and robustly express symmetry without explicit training of bidirectional stimulus pairings or spatiotemporal conditions that might promote it. Although the exact reasons behind the disparate proclivities with which humans and nonhumans express symmetry remain to be revealed, we hope that the present demonstrations of symmetry in humans can serve as a benchmark for future comparative efforts.

## AUTHOR CONTRIBUTIONS

Victor M. Navarro: Conceptualization, methodology, software, data curation, formal analysis, validation, investigation, visualization, writing original draft, review, and editing. Edward. A. Wasserman: Conceptualization, methodology, supervision, funding acquisition, project administration, resources, writing original draft, review, and editing.

## CONFLICT OF INTEREST STATEMENT

The authors have no conflicts of interest to disclose.

## ETHICS APPROVAL

All experimental procedures were according to the Declaration of Helsinki and were approved by the Institutional Review Board at the University of Iowa.

## Data Availability

All data and computer code used in the preparation of this work are available at https://osf.io/8qk4e/.

## Footnotes

^1^
The contingencies here assume select control by the reinforced stimuli (i.e., A in X|A + B‐ trials and B in Y|A‐B+ trials), but it is equally possible to observe symmetry under reject control by the nonreinforced stimuli (i.e., B in X|A + B‐ trials and A in Y|A‐B+ trials) or a mixture of both kinds of control, as pointed out by Sidman (1994).

^2^
An additional aspect of these networks is that they typically establish select control due to the 1 to 7 ratio of positive to negative comparisons per sample (e.g., Mandler, 1970). See Navarro (2020) for comparison‐replacement tests confirming that networks similar to these establish select control in pigeons and humans.

## APPENDIX A Instructions given to participants in Experiments 1 and 2

Thanks for agreeing to take part in this study!

In this study, you will be asked to complete a learning task.

On each screen, you will be shown a picture.

**Experiment 1 only:** [Then, two additional pictures will appear.]

**Experiment 2 only:** [Then, the picture will disappear, and two additional pictures will appear.]

Your task is to decide which one of the two pictures (left or right) is the correct picture based on the picture that appeared in the center of the screen.

If you think the picture on the left is correct, you should press the F key.

If you think the picture on the right is correct, you should press the J key.

You will receive feedback for your choice on every trial.

Your aim should be to make as many correct responses as you can.

You can receive a bonus payment, depending on how many correct responses you make.

We will collect data from a large number of participants in this task and calculate the average number of correct responses that they made.

If you make more correct responses than the average participant, you will receive a bonus of $3. So, the more correct responses you make, the more likely you are to receive a bonus.

There will be 480 trials in total. You can take as long as you like on each trial (your reaction time is not important in this study), but we recommend that you don't spend too long on any one trial. Typically, this experiment will take around 25–30 minutes to complete.

Please don't write anything down during the experiment.

## APPENDIX B Questions given to the participants after task instructions. Correct answers are underscored

Question 1: What will you be doing during this task?

- I will complete a questionnaire about my personality
- On each trial I will see a circle, and will be asked what color it is
- On each trial I will see three pictures, and will decide which of two side pictures is correct, based on the picture located in the center of the screen

Question 2: How often will you receive feedback on your decisions?

- Never
- After every trial
- After every 10 trials

Question 3: How can I receive a bonus of $3?

- I will receive a bonus if I make more correct responses than the average participant
- I will receive a bonus if more than half of my responses are correct
- I cannot receive a bonus
